# Supplementary material for: Genetic Profile and Toxigenic Potential of Bacillus cereus Isolates from a Norwegian Ice Cream Production Plant
Source: Foods. 2024 Sep 24;13(19):3029. doi: 10.3390/foods13193029 (PMC11475924; doi:10.3390/foods13193029)
Supplement: Supplementary file 1 [file foods-13-03029-s001.zip › foods-3217637-supplementary.pdf]

ID	Species	Date of isolation	Product	Production line	nheABC	cytK	hblCDA	Cereulide	Psychrotrofe/ Termophile	Sequence cluster
NVH-IS213	B. cereus	04.08.2021	a	1	+	-	-	-	NA	A
NVH-IS217	B. wiehenstephanensis	08.11.2021	b	2	+	-	+	-	P	
NVH-IS218	B. wiehenstephanensis	08.11.2021	b	2	+	-	+	-	P	
NVH-IS221	B. cereus	29.06.2021	b	2	+	-	-	-	T	
NVH-IS222	B. cereus	29.06.2021	b	2	+	-	-	-	NA	A
NVH-IS229	B. cereus	01.10.2021	c	2	+	-	+	-	P	B
NVH-IS230	B. cereus	01.10.2021	c	2	+	-	+	-	P	B
NVH-IS231	B. cereus	23.12.2021	c	2	+	+	+	-	T	
NVH-IS232	B. cereus	21.12.2021	Cream	3	+	-	+	-	P	B
NVH-IS233	B. cereus	21.12.2021	d	3	+	+	+	-	T	D
NVH-IS236	B. cereus	09.12.2021	e	3	+	-	-	-	T	
NVH-IS237	B. cereus	09.12.2021	e	3	+	+	+	-	T	
NVH-IS241	B. cereus	30.11.2021	f		+	+	+	-	T	
NVH-YM301	B. cereus	04.12.2022	g	1	+	-	+	-	T	C
NVH-YM302	B. cereus	04.12.2022	g	1	+	-	+	-	T	C
NVH-YM303	B. cereus	04.12.2022	g	1	+	-	-	+	T	
NVH-YM304	B. cereus	30.04.2022	g	1	+	-	-	-	T	
NVH-YM305	B. cereus	30.04.2022	g	1	+	-	-	-	T	
NVH-YM306	B. cereus	21.12.2021	c	2	+	+	-	-	T	
NVH-YM307	B. cereus	21.12.2021	c	2	+	+	+	-	T	F
NVH-YM308	B. cereus	21.12.2021	c	2	+	+	+	-	T	D
NVH-YM309	B. cereus	23.03.2022	c	2	+	+	+	-	T	D
NVH-YM311	B. cereus	14.01.2022	g	1	+	-	-	-	T	
NVH-YM312	B. cereus	14.01.2022	g	1	+	-	+	-	NA	
NVH-YM313	B. cereus	14.01.2022	g	1	+	-	-	-	T	
NVH-YM314	B. cereus	12.05.2022	h	2	+	-	-	-	T	
NVH-YM315	B. cereus	12.05.2022	h	2	+	-	-	-	T	
NVH-YM316	B. cereus	11.01.2022	c	2	+	-	-	-	NA	A
NVH-YM317	B. cereus	11.01.2022	c	2	+	-	-	-	NA	A
NVH-YM318	B. cereus	14.12.2021	h	2	+	-	-	-	T	
NVH-YM319	B. cereus	14.12.2021	h	2	+	+	+	-	T	F

NVH-YM320	B. cereus	10.01.2022	c	2	+	+	+	-	T	F
NVH-YM321	B. cereus	10.01.2022	c	2	+	+	+	-	T	D
NVH-YM322	B. cereus	09.12.2021	e	3	+	-	+	-	T	
NVH-YM324	B. cereus	22.12.2021	c	2	+	-	+	-	P	B
NVH-YM326	B. cereus	23.12.2021	c	2	+	+	+	-	P	B
NVH-YM327	B. cereus	23.12.2021	d	3	+	+	+	-	T	
NVH-YM328	B. cereus	23.12.2021	d	3	+	-	+	-	P	B
NVH-YM329	B. cereus	05.10.2022	i		+	+	+	-	NA	
NVH-YM330	B. cereus	04.10.2022	j		+	+	+	-	NA	
NVH-YM332	B. cereus	sep.22	Rework		+	-	-	-	NA	A
NVH-YM333	B. cereus	sep.22	Rework		+	+	+	-	T	
NVH-YM338	B. cereus	sep.22	Rework		+	+	+	-	T	
NVH-YM341	B. cereus	29.09.2022	k		+	+	+	-	T	
NVH-YM342	B. cereus	03.10.2022	Freezer	4	+	+	+	-	T	E
NVH-YM345	B. cereus	30.09.2022	l		+	-	-	-	T	
NVH-YM346	B. cereus	29.09.2022	Freezer	4	+	+	+	-	T	E
NVH-YM347	B. cereus	28.09.2022	Rework		+	-	-	-	NA	A
NVH-YM348	B. cereus	29.09.2022	Freezer	4	+	-	-	-	T	
NVH-YM349	B. cereus	28.09.2022	Pipeline		+	-	+	-	T	C
NVH-YM350	B. cereus	27.09.2022	Rework		+	-	+	-	T	C
NVH-YM351	B. cereus	27.09.2022	Rework		+	-	-	-	NA	A
NVH-YM353	B. cereus	28.09.2022	Freezer	4	+	+	+	-	T	E
NVH-YM355	B. cereus	27.09.2022	Freezer	5	+	-	-	-	T	
NVH-YM356	B. cereus	28.09.2022	Freezer	5	+	-	-	-	NA	A
NVH-YM357	B. cereus	27.09.2022	m		+	-	-	-	NA	A
NVH-YM359	B. cereus	27.09.2022	Cream		+	-	-	-	NA	A
BtB2	B. weihenstephanensis				+	-	+	+	P	
MC67	B. weihenstephanensis				+	-	+	+	P	
NC7401	B. cereus				+	-	-	+	M	
a-m	Type of product									
1 to 5	Line of production									
P	Psychrotrophic									
T	Termophilic									

NA	Not applicable								
A-F	Sequence cluster								
